# Effects of swimming training in hot and cold temperatures combined with cinnamon supplementation on HbA1C levels, TBC1D1, and TBC1D4 in diabetic rats

**DOI:** 10.1038/s41387-023-00256-0

**Published:** 2024-01-10

**Authors:** Seyed Morteza Tayebi, Amir Hossein Nouri, Bakhtyar Tartibian, Somayeh Ahmadabadi, Aref Basereh, Iman Jamhiri

**Affiliations:** 1 Associate Professor, Department of Exercise Physiology, Faculty of Sports Science, Allameh Tabataba’i University, Tehran, Iran; 2 MSc, Department of Exercise Physiology, Faculty of Sports Science, Allameh Tabataba’i University, Tehran, Iran; 3 Professor, Department of Exercise Physiology, Faculty of Sports Science, Allameh Tabataba’i University, Tehran, Iran; 4 Assistant Professor, Department of Physical Education and Sports Sciences, Farhangian University, Tehran, Iran; 5 Department of Exercise Physiology, Kharazmi University, Tehran, Iran; 6 Stem Cell Technology Research Center, Shiraz University of Medical Sciences, Shiraz, Iran

**Keywords:** Diabetes complications, Type 2 diabetes

## Abstract

**Aims:**

Diabetes is one of the main causes of mortality in developing countries. Performing physical activity in various ways and different environments using herbal supplements can be used as a non-pharmacological solution to prevent and improve diabetes. Hence, this study aimed to investigate the effects of eight weeks of cold water swimming exercise training combined with cinnamon supplementation on HbA1C (Hemoglobin A1c) levels, TBC1D1 (TBC1 domain family member 1), and TBC1D4 (TBC1 Domain Family Member 4) in diabetic rats.

**Materials and methods:**

Ninety-one rats (*n* = 78 diabetic, *n* = 13 healthy) were divided into seven groups (*n* = 13 per group): (1) healthy control (HC), (2) diabetic control (DC), (3) swimming training in cold water (5 °C) (S5), (4) swimming training in cold water (5 °C) with a cinnamon supplementation (200 mg/kg body weight) (S5+Ci), (5) swimming training in warm water (36-35 °C) (S35), (6) swimming training in warm water (35–36 °C) with a cinnamon supplementation (S35+Ci), and (7) a cinnamon supplementation only (Ci). To evaluate the hypothesis, a one-way ANOVA and Tukey’s post hoc test were used.

**Results:**

Findings showed that the TBC1D1 and TBC1D4 levels in the DC and S35 groups were higher than in the HC group (*p* < 0.001). Also, swimming training in cold water (5 °C) with cinnamon supplementation (S5+Ci) decreased the level of TBC1D1, TBC1D4, HbA1c, and glucose compared to other groups (*p* < 0.05).

**Conclusions:**

The study revealed that the combination of swimming training in cold water and cinnamon consumption led to a significant reduction in TBC1D1, TBC1D4, and HbA1c. Therefore, this non-traditional exercise approach coupled with cinnamon supplementation can be considered an effective method for improving insulin sensitivity, fasting blood glucose, and HbA1c levels and is proposed as an optimal method to improve glucose indices.

## Introduction

Diabetes is a widespread disorder affecting over 100 million people annually worldwide, with an estimated increase in prevalence from 171 million in 2000–366 million in 2030 [1, 2]. The increasing spread of diabetes and related diseases worldwide suggests that human progress in understanding the factors and mechanisms regulating body weight, particularly in preventing, combating, and treating diabetes, has not been very successful [3, 4].

As we reported recently, resistance training could decrease the glucose levels of people with diabetes, and improve insulin resistance, and it would be related to improved levels of meteorin-like protein (MTRNL) [5, 6]. We hypothesized that an increase in METRNL due to muscle contraction in line with intracellular calcium elevation increased the phosphorylation of TBC1D1 (Ser237) but inhibited TBC1D4 (Thr642) phosphorylation, indicating that METRNL stimulates the transportation and movement of GLUT4, specifically via AMPK-TBC1D1, so contribute to the improvement and treatment of insulin resistance [7].

The insulin signaling pathway relies heavily on Akt as a crucial point [8], serving as a point of divergence for downstream signaling to glucose transport via the TBC1 domain family, member 4 (TBC1D4), and TBC1 domain family, member 1 (TBC1D1), as well as to glycogen synthesis through glycogen synthase kinase 3 and glycogen synthase. TBC1D1 and TBC1D4 are 50% identical, and both contain a GAP (GTPase-activating Proteins) domain, two PTB (Phosphotyrosine-binding) domains, and a CBD (Calmodulin-binding Domain) domain [9]. TBC1D1 and TBC1D4 are regulated differently by specific upstream kinases in response to various stimuli such as insulin, muscle contraction, and AMPK (The AMP-activated protein kinase) [10, 11].

Several lines of evidence suggest that insulin, AMPK activation, and muscle contraction lead to an increase in phosphorylation and inactivation of TBC1D4 and TBC1D1, resulting in increased GTP loading onto Rab proteins on GLUT4 vesicles and increased translocation of GLUT4 to the plasma membrane. Thus, TBC1D1 and TBC1D4 regulate the communication pathways by insulin and glucose transport signals as new candidates [12]. In the active forms of TBC1D1 and TBC1D4, GTP hydrolysis to GDP on Rab proteins increases, inhibiting GLUT4 translocation [13].

Also, when blood glucose levels increase, the amount of glycated hemoglobin (A1C) increases, indicating poor blood glucose control, which may be associated with cardiovascular, neuropathic, nephropathic, and retinopathy diseases. Researchers found that changes in metabolic control in diabetic individuals are reflected more prominently in the assessment of hemoglobin A1C compared to glucose assessment [14].

Insulin and exercise are critical physiological stimuli for increasing glucose transport in skeletal muscle, and both cause glucose transporter 4 (GLUT4) redistribution from inside the cell to the cell surface membranes [15]. However, acute exercise and exercise training enhances subsequent insulin action on glucose transport [16, 17]. Since muscle contraction and insulin lead to TBC1D4 phosphorylation [18, 19], TBC1D4 is a potential point of convergence for exercise- and insulin-induced signaling to glucose uptake in human skeletal muscle [17, 18, 20].

Cold water immersion and cinnamon supplementation can also modulate glucose transport. Cold adaptation improves glucose tolerance and insulin sensitivity in mice with high-fat diets [21, 22], while cinnamon supplementation stimulates insulin receptor auto-phosphorylation and inhibits protein tyrosine phosphatase, an active enzyme in insulin receptor dephosphorylating, leading to increased insulin sensitivity [23]. Hanssen et al. [22] reported a decrease in plasma glucose concentration following cold acclimation in individuals with type 2 diabetes.

Therefore, since physical activity, cold water immersion, and cinnamon supplement consumption have shown beneficial effects in diabetic patients, further studies are needed to explore the therapeutic potential of these interventions in managing diabetes. Hence, this study aims to evaluate the effect of eight weeks of cold water swimming training combined with cinnamon supplementation on HbA1C levels, TBC1D1, and TBC1D4 in diabetic rats.

## Experimental section

### Animals

A total of 91 rats, aged 8 to 10 weeks, were procured from the animal breeding and reproduction center and acclimatized in the animal physiology laboratory for one week. The rats were maintained under standard conditions of 12 hours of darkness and 12 hours of light, 55% humidity, and a temperature of 22 to 24 °C. Transparent cages made of non-carbonate material with free access to food and water for rats. After one week, 78 rats were subjected to intraperitoneal injection of streptozotocin (Sigma, USA) at a dose of 55 mg/kg body weight, while a healthy control group (*n* = 13) was made to investigate the effect of inducing diabetes. Four days later, blood glucose levels of diabetic rats were measured by tail vein puncture, and diabetic rats were divided into six groups (*n* = 13 per group) for homogenization based on their fasting blood glucose levels [1]: Diabetic control (DC) [2], cold water swimming exercise (S5) [3], cold water swimming exercise with cinnamon extract supplementation (S5+Ci) [4], warm water swimming exercise (S35) [5], warm water swimming exercise with cinnamon supplementation (S35+Ci) [6], cinnamon supplementation (Ci), and [7] a Healthy control (HC).

## Experimental procedure

Initially, rats were trained and assessed for their swimming ability in cold water for one week, with daily sessions lasting 2 minutes. Throughout this acclimation period, the water temperature gradually decreased from 25 °C (the normal laboratory water temperature) to 5 °C. The rats’ activities were closely monitored for 2 minutes during each session until they attempted to escape from the situation. This process was repeated three times per week to familiarize the rats with the exercise conditions. Following this, swimming exercises were conducted at temperatures of 2 ± 5 °C and 2 ± 35 °C, according to the protocol by Lubkowska et al. [24], for 2 minutes a day, 5 days a week in the first week. An additional 30 seconds of exercise time was added to each session until the exercise duration reached 4 minutes. From then on, until the end of the eighth week, the rats swam for 4 minutes at a temperature of 5 °C. In the other group, the swimming exercise protocol at 35 °C was also performed concurrently. The rats swam in a specially designed swimming tank measuring 100 cm in length, 50 cm in width, and 50 cm in depth [24, 25].

### Cinnamon supplementation

Cinnamon extract was prepared by boiling 200 grams of dry cinnamon powder in 1000 ml of distilled water for 10 minutes and then filtered through a No. 1 filter paper after cooling. This solution contained 20% cinnamon extract, meaning that each milliliter of the solution contained 20 milligrams of cinnamon extract. For the cinnamon supplementation groups, 1 cc of this product was added to the drinking water for every 5 rats in the cage (approximately equivalent to 1 kilogram). Subsequently, the rats exercised for 8 weeks, with 5 sessions per week, using the provided exercise protocol. The rats in the cinnamon supplementation group received 200 milligrams of cinnamon extract per kilogram of body weight [26].

### Data collection

48 hours after the last training session, the rats were anesthetized using Xylazine ketamine, and their tissues were harvested. Blood samples were taken from the portal vein, and the plasma was collected by centrifugation at 3000 rpm for 10 minutes and sent to the laboratory for measurements. A1C was measured using the Pars Azmoon Glycosylated Hemoglobin (A1C) kit, an enzymatic method produced in Iran. The TBC1D1 level was measured using the enzyme-linked immunosorbent assay (ELISA) method with the TBC1D1 kit (sensitivity:100 pg/mL) produced by Zelbio in Germany, and the TBC1D4 level was measured using the ELISA method with the TBC1D4 kit (sensitivity:1000 ng/L) produced by Zelbio in Germany in the laboratory. According to the “Guide for the Care and Use of Laboratory Animals” prepared by the National Academy of Sciences and published by the National Institutes of Health (NIH publication 86-23 revised 1985), all animals were provided with compassionate care.

### Statistical analysis

The inferential statistical method of one-way analysis of variance (ANOVA) was used by the SPSS software for data analysis. Conducted the Homogeneity of variances and normality tests to ensure that the assumptions of ANOVA were met and Bonferroni post hoc test was used to investigate intergroup differences. Also, the Effect sizes were calculated by dividing the difference between the means pertaining to two groups by standard deviation. If the value is 0.2, it is considered a small effect, if it is 0.5, the effect is medium, and if 0.8 or more, it is a large effect. A significance level of less than 0.05 (*p* < 0.05) was considered acceptable.

## Result

### TBC1D4

**Fig. 1:**
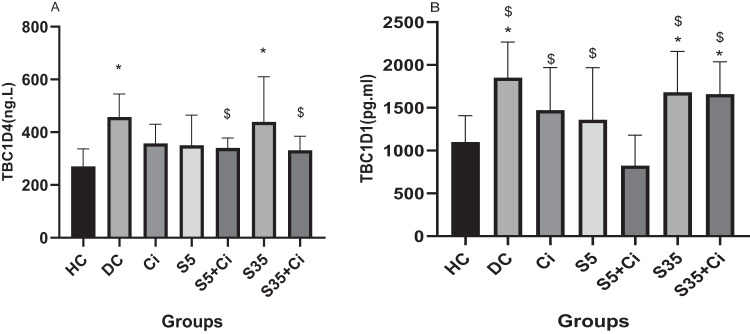
TBC1D4 and TBC1D1 levels in different groups. TBC1D4 level (**A**) and TBC1D1 level (**B**). Bars and whiskers represent the mean and SD, respectively. Significantly higher levels of TBC1D4 in DC and S35 groups (**A**). Significantly higher levels of TBC1D1 in DC, S35, and S35+Ci groups (**B**). Asterisk (*) indicates a significant difference compared to the HC group. The symbol $ indicates a significant difference compared to the S5+Ci group.

ANOVA revealed that the effect of groups was significant (*F*_(6,84)_ = 5.92, *p* < 0.001, *η*^2^ = 0.297). Bonferroni post hoc test results showed that the TBC1D4 level in the DC and S35 groups was higher than in the HC group (*p* < 0.001), and in the S5+Ci group was significantly lower than in the DC, S5, S35, and S35+Ci groups (*p* < 0.001). Other comparisons did not show significant differences (*p* > 0.05) (Fig. 1A).

### TBC1D1

ANOVA revealed that the effect of groups was significant (*F*_(6,84)_ = 8.51, *p* < 0.001, *η*² = 0.378). Bonferroni post hoc results showed that the TBC1D1 level in the DC, S35, and S35+Ci groups was higher than in the HC group (*p* < 0.001). Also, the TBC1D1 level of DC, Ci, S5, S35, and S35+Ci groups was significantly lower than the S5+Ci group (*p* = 0.037 and 0.019, respectively). Other comparisons did not show significant differences (*p* > 0.05) Fig. 1B).

### HbA1c

**Fig. 2:**
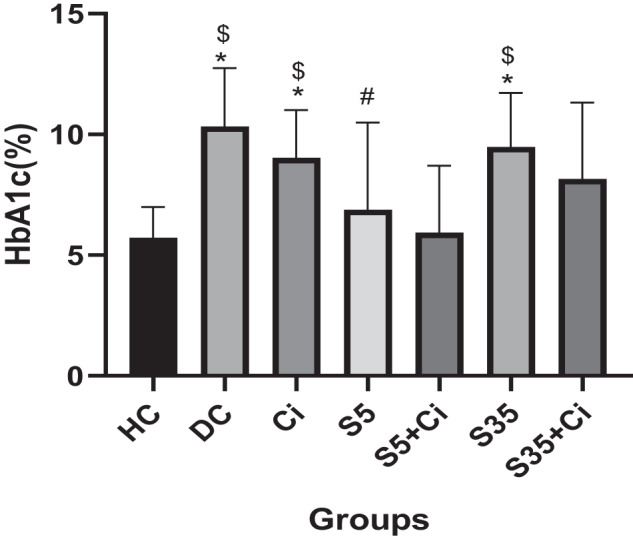
Mean ± SD of HbA1c in different groups. That (*) indicates a significant difference compared to the HC group. That ($) indicates a significant difference compared to the S5+Ci group.

ANOVA revealed that the effect of groups was significant (*F*_(6,84)_ = 6.25, *p* < 0.001, *η*² = 0.309). Bonferroni post hoc test results showed that the HbA1c level in the DC, Ci, and S35 groups was higher than in the HC group (*p* < 0.001), in the DC, Ci, and S35 groups was significantly lower than in the S5+Ci group (*p* < 0.001), and in the S5 group was significantly lower than in the DC group (*p* = 0.018). Other comparisons did not show significant differences (*p* > 0.05) Fig. 2).

## Discussion

The present study showed that diabetes and exercise in warm water (35 °C) increased TBC1D1 and TBC1D4 compared to the healthy group. Also, the present study is the first research that showed that swimming training in cold water (5 °C) with cinnamon supplementation decreased the level of TBC1D1 and TBC1D4 in diabetic rats. Also, the results of our study showed that swimming training in cold water with cinnamon supplementation decreased the blood glucose level in rats.

According to our review, no study was found to investigate the effects of cold and warm water temperatures on TBC1D1 and TBC1D4. Regarding the effect of temperature, some studies have shown that cold exposure is beneficial for glucose homeostasis. In this study, cold adaptation prevented the occurrence of reduced glucose tolerance and insulin sensitivity in rats that were fed a high-fat diet [21]. Despite this, various studies have investigated the long-term effects of TBC1D4. These studies showed that TBC1D1 and TBC1D4 phosphorylation levels increase [27–29] or remain unchanged after training [19, 30]. The 4 weeks of endurance training increased glucose uptake with no change in TBC1D4 in the hind limb muscles of rats fed an insulin-resistant diet [30]. In contrast, Vind et al. [27] suggested that the mechanisms by which exercise training improves insulin sensitivity in type 2 diabetes may involve augmented signaling of TBC1D4 and increased skeletal muscle content of key insulin signaling. TBC1D1 and TBC1D4 are involved in the regulation of muscle glucose transport. Overall, it has been suggested that exercise training induces greater phosphorylation of TBC1D1 and TBC1D4 in insulin-stimulated muscles, which, together with other effects of exercise training (such as increased TBC1D1 and TBC1D4 protein expression) may contribute to improved insulin sensitivity [31].

Other findings of this research showed a significant decrease in HbA1c in the two groups: swimming in cold water and swimming in cold water with consumption of cinnamon, compared to other groups. In line with our findings, most of the conducted studies have shown a significant reduction in HbA1c after different training sessions in healthy people and those with type 2 diabetes [5, 32–36]. The most recent review and meta-analysis conducted by Liu and colleagues indicated that high-intensity Resistance training was more effective than low-to-moderate-intensity in reducing HbA1c [37]. Also, the results of our study showed that the rate of HbA1c increases with the rise in water temperature, which is in line with the results of other studies [38, 39]. Alghamdi et al. [40] showed that a rise in ambient temperature is associated with increased HbA1c, which could harm the health of people suffering from diabetes. Possible reasons for an increase in HbA1c could include reduced physical activity [40, 41], decreased sunlight exposure, and dehydration during hot weather [38]. No research was found regarding the effect of exercise in water with different temperatures on HbA1c, and this study, exercise interventions in different water temperatures, can be a new topic for further research.

In our findings, swimming in cold water along with cinnamon supplementation caused a significant decrease in the levels of TBC1D1 and TBC1D4. Also, consumption of cinnamon alone and along with swimming exercises in cold water caused a significant decrease in HbA1c. In line with current results, most studies reported improvement in glucose indices regarding cinnamon consumption alone or with exercise [42]. Mechanisms of a reduction in TBC1D1 and TBC1D4 after consuming cinnamon alone or in different temperatures have not been identified according to our investigations, and no study was found. However, contradictory studies were observed related to HbA1c [43–47]. In a study of 5 prospective-controlled trials by Baker et al., cinnamon supplementation was found to not alter HbA1c in patients with type 1 and type 2 diabetes [42]. In contrast, in the study of Crawford et al., 109 type 2 diabetes mellitus (HbA1c > 7) patients were evaluated for 90 days. It was found that the daily consumption of 1 g of cinnamon capsules significantly reduced the HbA1c level [48]. It has been shown that cinnamon extract reduces blood glucose levels and improves insulin resistance in rats by increasing insulin activity and glucose metabolism in fat cells [49].

Cinnamon, by inhibiting the insulin receptor phosphatase enzyme, activates the insulin receptor-kinase enzyme in fat cells. This leads to a decrease in insulin resistance and an increase in the sensitivity of these cells to insulin. Consequently, some research has linked the reduction of HbA1c to the mechanisms mentioned above after consuming cinnamon [50, 51].

As no study has been conducted on the impact of different temperatures on the variables of our study to understand the mechanisms involved, we can refer to the previous studies on the effect of temperature on insulin sensitivity and GLUT4. The changes in TBC1D1, TBC1D4, and HbA1c may be related to changes in insulin sensitivity or GLUT4. However, further research is necessary to fully investigate the mechanisms involved.

## Conclusion

This study found that after 8 weeks of swimming training in cold water combined with cinnamon consumption, there was a significant reduction in TBC1D1 and TBC1D4 compared to other groups. Additionally, both groups (S5, S5+Ci) that swam in cold water experienced a significant decrease in HbA1c levels. The results suggest that exercising in a cold environment can improve insulin sensitivity, and HbA1c levels. Therefore, swimming in cold water with cinnamon consumption may be a beneficial alternative exercise method in comparison with traditional approaches for improving glucose indices.

## Data Availability

All data generated or analyzed during this study are included in this published article.

## References

[1] Vanschoonbeek K, Thomassen BJ, Senden JM, Wodzig WK, van Loon LJ (2006). Cinnamon supplementation does not improve glycemic control in postmenopausal type 2 diabetes patients. The J Nutr.

[2] Saeidi A, Hackney AC, Tayebi SM, Ahmadian M, Zouhal H (2019). Diabetes, insulin resistance, fetuin-B and exercise training. Ann Appl Sport Sci.

[3] Saeidi A, Tayebi SM, Khosravi A, Razi O, Sellami M, Abderrahman AB (2019). Obesity, fat mass, osteopontin and exercise training. Int J Appl Exerc Physiol.

[4] Tayebi SM, Ghanbari-Niaki A, Saeidi A, Hackney AC (2017). Exercise training, neuregulin 4 and obesity. Ann Appl Sport Sci.

[5] Tayebi SM, Golmohammadi M, Eslami R, Shakiba N, Costa PB (2023). The effects of eight weeks of circuit resistance training on serum METRNL levels and insulin resistance in individuals with type 2 diabetes. J Diabetes Metab Disord.

[6] Saeidi A, Tayebi SM, Khosravi A, Malekian F, Khodamoradi A, Sellami M (2019). Effects of exercise training on type 2-diabetes: the role of Meteorin-like protein. Health Promot Perspect.

[7] Lee JO, Byun WS, Kang MJ, Han JA, Moon J, Shin MJ (2020). The myokine meteorin‐like (metrnl) improves glucose tolerance in both skeletal muscle cells and mice by targeting AMPKα2. FEBS J.

[8] Taniguchi CM, Emanuelli B, Kahn CR (2006). Critical nodes in signalling pathways: insights into insulin action. Nat Rev Mol Cell Biol.

[9] Roach WG, Chavez JA, Mîinea CP, Lienhard GE (2007). Substrate specificity and effect on GLUT4 translocation of the Rab GTPase-activating protein Tbc1d1. Biochem J.

[10] An D, Toyoda T, Taylor EB, Yu H, Fujii N, Hirshman MF (2010). TBC1D1 regulates insulin-and contraction-induced glucose transport in mouse skeletal muscle. Diabetes.

[11] Tobias IS, Lazauskas KK, Siu J, Costa PB, Coburn JW, Galpin AJ (2020). Sex and fiber type independently influence AMPK, TBC1D1, and TBC1D4 at rest and during recovery from high-intensity exercise in humans. J Appl Physiol.

[12] Kahn BB, Alquier T, Carling D, Hardie DG (2005). AMP-activated protein kinase: ancient energy gauge provides clues to modern understanding of metabolism. Cell Metab.

[13] Sakamoto K, Holman GD (2008). Emerging role for AS160/TBC1D4 and TBC1D1 in the regulation of GLUT4 traffic. Am J Physiol Endocrinol Metab.

[14] Larsen RN, Mann NJ, Maclean E, Shaw J (2011). The effect of high-protein, low-carbohydrate diets in the treatment of type 2 diabetes: a 12 month randomised controlled trial. Diabetologia.

[15] Richter EA, Hargreaves M. Exercise, GLUT4, and skeletal muscle glucose uptake. Physiol Rev. 2013;93:993–17. 10.1152/physrev.00038.2012 23899560

[16] Wojtaszewski JF, Richter EA (2006). Effects of acute exercise and training on insulin action and sensitivity: focus on molecular mechanisms in muscle. Essays Biochem.

[17] Frøsig C, Rose AJ, Treebak JT, Kiens B, Richter EA, Wojtaszewski JF (2007). Effects of endurance exercise training on insulin signaling in human skeletal muscle: interactions at the level of phosphatidylinositol 3-kinase, Akt, and AS160. Diabetes.

[18] Treebak JT, Frøsig C, Pehmøller C, Chen S, Maarbjerg SJ, Brandt N (2009). Potential role of TBC1D4 in enhanced post-exercise insulin action in human skeletal muscle. Diabetologia.

[19] Treebak JT, Birk JB, Rose AJ, Kiens B, Richter EA, Wojtaszewski JF (2007). AS160 phosphorylation is associated with activation of α2β2γ1-but not α2β2γ3-AMPK trimeric complex in skeletal muscle during exercise in humans. Am J Physiol Endocrinol Metab.

[20] Cartee GD, Wojtaszewski JF (2007). Role of Akt substrate of 160 kDa in insulin-stimulated and contraction-stimulated glucose transport. Appl Physiol Nutr Metab.

[21] Vallerand AL, Lupien J, Bukowiecki LJ (1986). Cold exposure reverses the diabetogenic effects of high-fat feeding. Diabetes.

[22] Hanssen MJ, Hoeks J, Brans B, Van Der Lans AA, Schaart G, Van Den Driessche JJ (2015). Short-term cold acclimation improves insulin sensitivity in patients with type 2 diabetes mellitus. Nat Med.

[23] Anderson RA (2008). Chromium and polyphenols from cinnamon improve insulin sensitivity: plenary lecture. Proceed Nutr Soc.

[24] Lubkowska A, Bryczkowska I, Gutowska I, Rotter I, Marczuk N, Baranowska-Bosiacka I (2019). The effects of swimming training in cold water on antioxidant enzyme activity and lipid peroxidation in erythrocytes of male and female aged rats. Int J Environ Res Public Health.

[25] Bryczkowska I, Baranowska-Bosiacka I, Lubkowska A (2017). Effect of repeated cold water swimming exercise on adaptive changes in body weight in older rats. Central Eur J Sport Sci Med.

[26] Badalzadeh R, Shaghaghi M, Mohammadi M, Dehghan G, Mohammadi Z (2014). The effect of cinnamon extract and long-term aerobic training on heart function, biochemical alterations and lipid profile following exhaustive exercise in male rats. Adv Pharm Bull.

[27] Vind B, Pehmøller C, Treebak JT, Birk JB, Hey-Mogensen M, Beck-Nielsen H (2011). Impaired insulin-induced site-specific phosphorylation of TBC1 domain family, member 4 (TBC1D4) in skeletal muscle of type 2 diabetes patients is restored by endurance exercise-training. Diabetologia.

[28] Kjøbsted R, Roll JL, Jørgensen NO, Birk JB, Foretz M, Viollet B (2019). AMPK and TBC1D1 regulate muscle glucose uptake after, but not during, exercise and contraction. Diabetes.

[29] Kido K, Ato S, Yokokawa T, Makanae Y, Sato K, Fujita S (2016). Acute resistance exercise‐induced IGF 1 expression and subsequent GLUT 4 translocation. Physiol Rep.

[30] Brandt N, De Bock K, Richter EA, Hespel P (2010). Cafeteria diet-induced insulin resistance is not associated with decreased insulin signaling or AMPK activity and is alleviated by physical training in rats. Am J Physiol Endocrin Metab.

[31] Cartee GD (2015). Roles of TBC1D1 and TBC1D4 in insulin-and exercise-stimulated glucose transport of skeletal muscle. Diabetologia.

[32] Gholami M, Eftekhari E, Zafari A, Solatzadeh O (2017). Effect of eight weeks low and moderate intensity aerobic training on levels of HbA1C, some hematological parameters and percent body fat in overweight and obese men with type 2 diabetes. Metab Exerc.

[33] Ghafari M, Faramarzi M, Hemati Farsani Z (2020). Effect of endurance training with different intensities on HBA1C in type 2 diabetes. Armaghane Danesh.

[34] Jansson AK, Chan LX, Lubans DR, Duncan MJ, Plotnikoff RC (2022). Effect of resistance training on HbA1c in adults with type 2 diabetes mellitus and the moderating effect of changes in muscular strength: a systematic review and meta-analysis. BMJ Open Diabetes Res Care.

[35] Tayebi SM, Eslami R, Iranshad I, Golmohammadi M (2023). The effect of eight weeks of circuit resistance training on serum levels of GPR119 and β-arrestin1 in individuals with type 2 diabetes. Ann Appl Sport Sci.

[36] Tayebi SM, Saeidi A, Shahghasi R, Golmohammadi M (2023). The eight-week circuit resistance training decreased the serum levels of WISP-1 and WISP-2 in individuals with type 2 diabetes. Ann Appl Sport Sci.

[37] Liu Y, Ye W, Chen Q, Zhang Y, Kuo C-H, Korivi M (2019). Resistance exercise intensity is correlated with attenuation of HbA1c and insulin in patients with type 2 diabetes: a systematic review and meta-analysis. Int J Environ Res Public Health.

[38] Alghamdi AS, Alqadi A, Alghamdi F, Jenkins RO, Haris PI (2021). Higher ambient temperature is associated with worsening of HbA1c levels in a Saudi population. Int J Clin Exp Pathol.

[39] Tien K-J, Yang C-Y, Weng S-F, Liu S-Y, Hsieh M-C, Chou C-W (2016). The impact of ambient temperature on HbA1c in Taiwanese type 2 diabetic patients: the most vulnerable subgroup. J Formos Med Assoc.

[40] Tayebi SM, Hasannezhad P, Saeidi A, Fadaei MR (2018). Intense circuit resistance training along with zataria multiflora supplementation reduced plasma retinol binding protein-4 and tumor necrosis factor-α in postmenopausal females. Jundishapur J Nat Pharm Prod.

[41] Tayebi SM, Saeidi A, Fashi M, Pouya S, Khosravi A, Shirvani H (2019). Plasma retinol-binding protein-4 and tumor necrosis factor-α are reduced in postmenopausal women after combination of different intensities of circuit resistance training and Zataria supplementation. Sport Sciences for Health.

[42] Baker WL, Gutierrez-Williams G, White CM, Kluger J, Coleman CI (2008). Effect of cinnamon on glucose control and lipid parameters. Diabetes Care.

[43] Gupta Jain S, Puri S, Misra A, Gulati S, Mani K (2017). Effect of oral cinnamon intervention on metabolic profile and body composition of Asian Indians with metabolic syndrome: a randomized double-blind control trial. Lipids Health Dis.

[44] Talaei B, Amouzegar A, Sahranavard S, Hedayati M, Mirmiran P, Azizi F (2017). Effects of cinnamon consumption on glycemic indicators, advanced glycation end products, and antioxidant status in type 2 diabetic patients. Nutrients.

[45] Zare R, Nadjarzadeh A, Zarshenas MM, Shams M, Heydari M (2019). Efficacy of cinnamon in patients with type II diabetes mellitus: a randomized controlled clinical trial. Clin Nutr.

[46] Kizilaslan N, Erdem NZ (2019). The effect of different amounts of cinnamon consumption on blood glucose in healthy adult individuals. Int J Food Sci.

[47] Lira Neto JCG, Damasceno MMC, Ciol MA, de Freitas RWJF, de Araújo MFM, Teixeira CRDS (2022). Efficacy of cinnamon as an adjuvant in reducing the glycemic biomarkers of type 2 diabetes mellitus: a three-month, randomized, triple-blind, placebo-controlled clinical trial. J Am Nutr Assoc.

[48] Crawford P (2009). Effectiveness of cinnamon for lowering hemoglobin A1C in patients with type 2 diabetes: a randomized, controlled trial. J Am Board Fam Med.

[49] Cao H, Polansky MM, Anderson RA (2007). Cinnamon extract and polyphenols affect the expression of tristetraprolin, insulin receptor, and glucose transporter 4 in mouse 3T3-L1 adipocytes. Arch Biochem Biophys.

[50] Mirfeizi M, Mehdizadeh Tourzani Z, Mirfeizi SZ, Asghari Jafarabadi M, Rezvani H, Shoghi M (2014). Effects of cinnamon on controlling blood glucose and lipids in patients with type II diabetes mellitus: a double blind, randomized clinical trial. Clin Nutr..

[51] Zahedifar A, Khodashenas M, Bijari B, Zahedifar F (2018). Effects of cinnamon on fasting blood sugar and hemoglobin A1C in patients with type II diabetes mellitus: a randomized clinical trial. J Maz Univ Med.

